# Meibomian gland dysfunction: hyperkeratinization or atrophy?

**DOI:** 10.1186/s12886-015-0132-x

**Published:** 2015-12-17

**Authors:** James V. Jester, Geraint J. Parfitt, Donald J. Brown

**Affiliations:** Gavin Herbert Eye Institute, University of California, Irvine, CA USA

**Keywords:** Meibomian gland dysfunction, Evaporative dry eye disease, Ocular surface, 3-D reconstruction, Immunofluorescence

## Abstract

Meibomian gland dysfunction (MGD) is the major cause of evaporative dry eye disease (EDED) and dysfunction is widely thought to mechanistically involve ductal hyperkeratinization, plugging and obstruction. This review re-evaluates the role of hyperkeratinization in MGD based on more recent findings from mouse models. In these studies, eyelids from normal young and old mice or mice exposed to desiccating stress were evaluated by immunofluorescent tomography and 3-dimensional reconstruction to evaluate gland volume, expression of hyperkeratinization markers and cell proliferation or stimulated Raman scattering (SRS) microscopy to assess lipid quality. Results indicate that aging mice show dropout of meibomian glands with loss of gland volume and a forward migration of the mucocutaneous junction anterior to the gland orifice; similar age-related changes that are detected in human subjects. Atrophic glands also showed evidence of epithelial plugging of the orifice without the presence of hyperkeratinization. Mice exposed to desiccating stress showed hyperproliferation of the meibomian gland and ductal dilation suggesting a marked increase in lipid synthesis. Lipid quality was also affected in EDED mice with an increase in the protein content of lipid within the duct of the gland. Overall, age-related changes in the mouse show similar structural and functional correlates with that observed in clinical MGD without evidence of hyperkeratinization suggesting that gland atrophy may be a major cause of EDED. The response of the meibomian gland to desiccating stress also suggest that environmental conditions may accelerate or potentiate age-related changes.

## Introduction

Meibomian glands are modified, holocrine, sebaceous glands that are embedded in the tarsal plate of the both the upper and lower eyelid [1], and excrete lipid onto the surface of the eye to form the lipid layer of the tear film to reduce aqueous tear evaporation [2]. Dysfunction of the meibomian gland (MGD) is a common eyelid disorder having a widespread prevalence of 39–50 % in the US population with the incidence increasing with age [3–6]. MGD is also a major cause of evaporative dry eye disease (EDED) [7], with loss of glands resulting in decreased tear film lipid, increased aqueous tear evaporation [2], and increased tear film osmolarity [8]; leading to ocular surface changes, unstable tear film and blepharitis [9, 10]. While patients with EDED and MGD comprise from 37 to 47 % of the average Ophthalmologists and Optometrists practice, management of this disease is primarily palliative and includes warm compresses, anti-microbial and anti-inflammatory therapy [4].

Currently, three forms of MGD are recognized: hypersecretory MGD, hyposecretory MGD and obstructive MGD, with the later form considered to be the most common [11, 12]. Based on clinical and animal studies [13–19], obstructive MGD is thought to involve hyperkeratinization of the meibomian gland duct leading to ductal occlusion and plugging of the meibomian gland orifice that then causes cystic dilation of the duct and a ‘disuse atrophy’ of the acini that is detected as gland ‘dropout’ on transillumination infrared photography (meibography) [20].

Recent studies of human and mouse meibomian glands have identified specific age-related changes including decreased acinar cell proliferation, gland atrophy and altered peroxisome proliferator-activated receptor gamma (PPARγ) expression and localization [21, 22]. Since PPARγ is a major regulator of lipogenesis and is required for sebocyte and adipocyte differentiation [23], these findings suggest that during aging there is a decline in meibocyte differentiation and lipid synthesis that leads to an age-related meibomian gland dysfunction (ARMGD) causing meibomian gland dropout and abnormal lipid excretion. More recent studies evaluating meibomian gland function in the mouse further support a role for meibomian gland atrophy as a potential major cause for clinical MGD and EDED. These experimental findings are inconsistent with the conventional theory of hyperkeratinization and duct obstruction as the mechanistic basis for MGD. This review presents the original evidence for keratinization playing a role in the development of obstructive MGD as well as discuses recently published findings on keratinization in ARMGD and the effects of desiccating stress on gland function. Based on this review, we hypothesize that defects in meibomian gland acinar differentiation and function leading to gland atrophy play a critical role in the development of clinical MGD as opposed to a mechanism involving hyperkeratinization leading to duct obstruction.

### Hyperkeratinization and meibomian gland dysfunction

In 1979, while studying a non-human primate model of polychlorinated biphenyl (PCB) poisoning in human, Ohnishi et al. showed that the ocular manifestations of this disease was associated with hyperkeratosis of the meibomian gland duct leading to ductal dilation and filling with keratinized cells [18]. Since it was generally believed at that time that obstruction and dilation of the meibomian gland did not involve keratinization [24], it was suggested by Ohnishi that keratic cyst formation was a characteristic of PCB poisoning. Later, in 1980 and 1981, Korb and Henriquez published their findings on contact lens intolerance and the association with MGD involving deficient secretions leading to ocular dryness and fluorescein staining of the cornea [19, 25]. In this study, meibomian gland secretions were collected from contact lens intolerant patients and age and sex matched contact lens tolerant patients and evaluated by cytological staining. Unlike samples from contact lens tolerant patients, they observed that contact lens intolerant patients showed keratotic plugging of the meibomian glands and the presence of desquamated epithelial cells in the stagnated excretions taken from the glands. As a mechanistic basis for their findings, they proposed that internal obstruction and plugging of the meibomian gland duct may be related to increased epithelial turnover and the accumulation of desquamated epithelial cells, particularly near the lid margin.

Later in 1981, a histologic and ultrastructural study of the human, primate, rabbit and steer meibomian gland was published showing that the ductal epithelium of the gland exhibited features similar to keratinized epidermis, including a thin horny layer, keratohyaline granules and lamellar bodies; albeit a well developed stratum corneum and stratum granulosum were absent except at the orifice of the gland [1]. These structural features bore distinct similarities to that of the sebaceous gland suggesting that similar pathogenic mechanisms involving keratinization may play a role in meibomian gland disease.

**Fig. 1:**
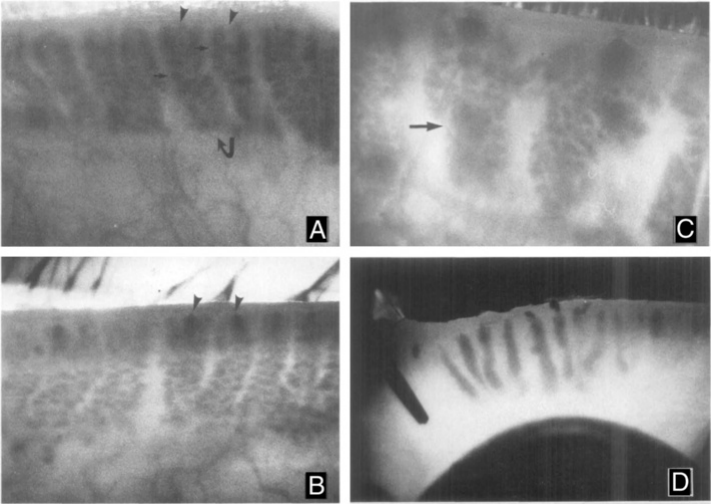
Meibography of the rabbit eyelids from normal (**a**), and 2 % epinephrine treated eyes for 1–2 months (**b**), 3–4 months (**c**) and 6 months (**d**). Normal glands (**a**. *curved arrow*) appear as grape-like clusters of individual acini (*small arrow*) with indistinct gland orifices (**a**, *arrowheads*). After 1–2 months of treatment, the orifices of the glands become detectible as dark spots at the leading edge of the glands (**b**, *arrowheads*). Progression of hyperkeratinization into the gland is detected by 3–4 months after treatment as large, dark cystic structure that obscure the normal acini (**c**, *arrows*). After six months of treatment the meibomian glands are replaced by large, dense keratic cysts (**d**). Magnification A-C: 24x and D: 10x

In 1982, a rabbit model of meibomian gland hyperkeratinization following long term topical epinephrine treatment was reported along with a novel imaging approach to sequentially document the progression of disease using transillumination biomicroscopy, i.e., meibography [16]. As shown in Fig. 1, meibography as originally described by Tapie [26], detects primarily the individual acini of the meibomian glands as they cluster around the central duct (a, small arrows). In normal eyelids, the orifice of the glands remains indistinct (a, arrowheads), while rabbits eyes treated with 2 % epinephrine for 1–2 months showed marked darkening in the region of the gland orifice (b, arrowheads). On histological evaluation, the dark regions identified by meibography were associated with hyperkeratinization and plugging of the orifice of the gland and the beginning of ductal dilation. Continued exposure to 2 % epinephrine resulted in progressive hyperkeratinization of the gland and the appearance of dark, cystic structures that obscured the normal appearance of the gland and was consistent with the beginning of acinar atrophy and replacement with fully keratinized epithelium. Long-term treatment resulted in complete replacement of the gland with a dark keratotic cyst as seen by meibography (D). Of particular note in this study was the finding that ductal plugging by hyperkeratinzation was first detected using meibography by the distinct darkening at the orifice of the gland.

Also in 1982, Gutgesell et al. reported on the histologic findings from 7 patients exhibiting MGD who underwent ectropion or entropion repair [13]. Pathologic samples showed dilation of the central meibomian gland duct with evidence of acinar atrophy and the presence of regional "hyperkeratinization" or thickening of the ductal epithelium. Together these findings along with other confirmatory reports have formed the basis for a putative pathogenic mechanism for MGD involving hyperkeratinization of the meibomian gland ductal epithelium leading to obstruction of the meibomian gland orifice, stasis of the gland, cystic dilation and then atrophy of the secretory acini as extensively summarized by the recent International Workshop on Meibomian Gland Dysfunction [12]. While this model is widely accepted, there are several observations in patients with MGD that are not explained by hyperkeratinization.

### Inconsistencies in the hyperkeratinization model

**Fig. 2:**
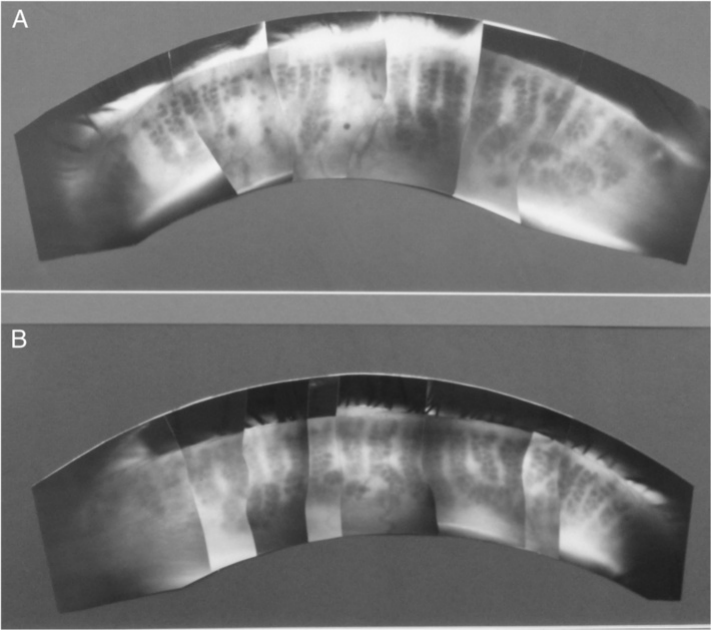
Meibography of a patient suffering from chronic blepharitis (**a**) showing extensive meibomian gland acinar dropout compared to a normal individual (**b**)

As shown in Fig. 2, the hallmark of MGD is dropout of the acini within the eyelid of patients suffering chronic blepharitis compared to normal individuals [27]. In general, the severity of meibomian gland dropout coincides with decrease quality of the meibomian gland excreta, showing a significantly high correlation (r = 0.882, *p* < 0.001) in one reported study of 36 sequential oculoplastic patients [21]. Intuitively, based on the hyperkeratinization model one might expect a much lower correlation if changes in lipid quality and increased excreta viscosity following duct obstruction preceded ductal dilation and acinar atrophy. Such is the apparent case for PCB poisoning, where hypersecretion of a whitish substance easily expressed from the meibomian glands is one of the first ocular symptoms detected in patients and non-human primates treated for 1 month with PCB [18].

Another marked difference between animal models of hyperkeratinization induced MGD and clinical MGD is the absence of any meibographic changes in the orifice of the gland in patients. Unlike the rabbit epinephrine-induced MGD model, where the first signs of ductal obstruction can be detected by focal darkening at the gland orifice [16], similar changes are not observed in chronic blepharitis patients [27]. Furthermore, while progressive keratinization of the meibomian gland in the rabbit is detected by the development of dark cystic structures that obscure acinar detail, chronic blepharitis patients show simply continued loss of acini. Even more recent publications using non-invasive and non-contact imaging systems, analysis of MGD continues to focus on gland dropout, the severity of which has been shown to be highly correlated with meibomian gland lipid changes [28, 29].

While these differences could be species specific, the biochemical signatures between the hyperkeratinization rabbit model and chronic blepharitis associated MGD are also very different. In rabbits, cytokeratin proteins characteristic of fully keratinized, skin epidermis (65–67 kD) are not detected in the excreta from normal eyelids, or eyelids not showing meibographic evidence of epinephrine-induced MGD [14, 30]. However, these cytokeratin markers for keratinization can be detected in excreta from rabbits showing ductal plugging, and are present in increasing amounts as hyperkeratinization of the glands progress. By contrast, evaluation of cytokeratins in excreta from MGD patients failed to detect keratin markers for hyperkeratinization [31]. Although MGD excreta contained a 10 % increase in the amount of detectible cytokeratins, these were associated with ductal keratins and not fully keratinized epithelia. The authors suggest that the failure to detect keratinization markers could be due to increase susceptibility to degradation of higher molecular weight keratin, a similar explanation for the absence of these markers in normal excreta. While keratinization markers have been detected using proteomic analyses [32], it should be noted that MGD patients do not show meibographic evidence of ductal plugging similar to rabbits, and therefore the failure to detect keratinization markers on immunoblotting is consistent with the rabbit findings. Although it is clear that Korb and Henriquez identified squamous cells within excreta from MGD patients [25], it is possible that these cells were not fully keratinized (a claim not made by Korb and Henriquez) and were rather derived from non-keratinized ductal epithelial cells.

### Age-related meibomian gland dysfunction and keratinization

Recently, an age-related meibomian gland dysfunction (ARMGD) leading to acinar dropout and significantly decreased gland size has been identified in aging mice [22, 33]. To evaluate the role of gland keratinization in ARMGD, immunofluorescent tomography (IT) has been used to localize and quantify changes in keratinization markers, comparing young mice (5 month-old) to older mice (2 year-old) [34]. IT is a novel imaging paradigm that uses plastic embedding, serial thin sectioning of large tissue blocks (3 mm × 3 mm) and repetitive immunofluorescent staining of individual sections to 3-dimensionally reconstruct at high resolution (voxels = 0.44 μm × 0.44 μm × 2.0 μm) large portions of the mouse eyelid containing multiple meibomian glands (for details please see reference [35]).

**Fig. 3:**
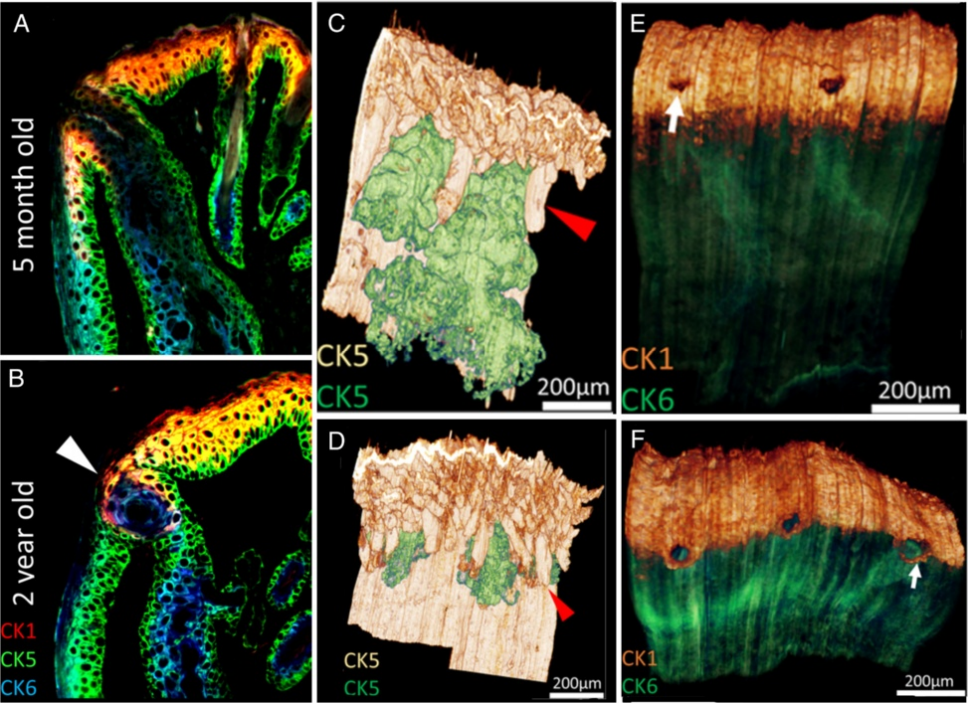
Immunofluorescent Tomography of eyelids from 5 month-old (**a**, **c**, **e**) and 2 year-old (**b**, **d**, **f**) mice. In single plastic sections (**a** and **b**) sequentially stained for cytokeratin 1 (CK1, red), 5 (CK5, green) and 6 (CK6, blue) the mucocutaneous junction is detected by the transition from fully keratinized skin (CK1 positive) to non-keratinized conjunctiva (CK6 positive). Note the presence of ductal plugging with CK6 positive epithelial cells in the 2 year-old eyelid (*arrowhead*). Volume reconstruction of anti-CK5 staining of eyelids (**c** and **d**) with segmentation of the meibomian glands (*green*) from skin epithelium (white) shows marked atrophy of the meibomian glands in the 2 year-old compared to the 5 month-old eyelid (*red arrowhead*). Reconstruction of young and old eyelids (**e** and **f**) stained for CK1 epidermis (*gold*) and CK6 conjunctiva (*green*) show the anterior migration of the mucocutaneous junction (**e** and **f**, arrows)

As shown in Fig. 3, individual sections can be sequentially stained to localize multiple cytokeratins and determine where in the meibomian gland cytokeratin markers for fully keratinized epithelium (CK1) compared to markers for non-keratinized epithelium (CK6) are expressed. In young mice (A), CK1 staining extends from the fully keratinized epidermis into the ductal epithelium at the orifice and then extends posteriorly toward the conjunctiva. CK1 staining abruptly stops where CK6 staining begins, which is posterior to the gland orifice in young mice; indicating the location of the mucocutaneous junction or the region where fully keratinized skin epidermis meets non-keratinized conjunctiva. Importantly, it should also be noticed that in young mice the suprabasal ductal epithelium of the meibomian gland is stained by CK6 similar to conjunctiva, indicating that while ultrastructurally there are features suggestive of full keratinization, ductal epithelium is non-keratinized. Similar cytokeratin staining patterns in the mouse have been reported by others [36].

In tissue sections from older mice (b), CK1 staining appears to stop at the orifice of the gland (arrowhead), suggesting an anterior shift in the mucocutaneous junction. Additionally, in some glands the orifice appeared plugged with epithelial cells that stained with antibodies to CK6 and not CK1 (b, arrowhead). 3-Dimensional reconstructions confirmed the large size of the meibomian glands in young mice and the marked decrease in size with aging mice (red arrowhead, c and d, respectively). Additionally, surface rendering for CK1 (e and f, gold) combined with CK6 (e and f, green) more clearly identified the location of the mucocutaneous junction posterior to the meibomian gland orifice in young mice (e). By contrast, the mucocutaneous junction had moved anteriorly in older mouse eyelids and in some cases did not surround the entire orifice (f, arrow).

There are two very interesting observations regarding ARMGD in the mouse that are inconsistent with the proposed mechanism of hyperkeratinization, ductal obstruction and MGD. First, epithelial plugs were detected at the orifice of some of the glands that appear to be comprised of non-keratinized ductal epithelial cells. The presence of epithelial plugging is consistent with the observations by Korb and Henriquez that excreta from patient with MGD contain desquamated cells and at times epithelial ductal plugs [19]. However, since Korb and Henriquez did not specifically stain for the presence of keratinization, it is not clear that the epithelial ductal plugs that were observed represented "keratotic plugs" as described in their report. The finding that plugs were comprised of non-keratinized ductal epithelium also supports the observations by Ong et al. that excreta from MGD patients does not contain cytokeratins characteristic of fully keratinized skin [31], and is not what is observed in hyperkeratinization models of MGD [30].

Secondly, ARMGD in the mouse is associated with an anterior displacement of the mucocutaneous junction. A very similar anterior displacement is detected in aging humans as identified by fluorescein staining of Marx line [37]. More importantly, the report by Yamaguchi et al. indicates that there is a very strong correlation with the position of Marx line and both meibomian gland dropout and abnormal meibomian gland secretion. While it is not clear whether the development of MGD precedes or is caused by this forward displacement, it is clear that ARMGD in the mouse shows distinctly similar structural changes in the mucocutaneous junction as observed in aging subjects and that meibomian gland dropout and anterior displacement of the mucocutaneous junction are likely closely related. Another, and perhaps more important point that can be deduced from these findings is that hyperkeratinization does not play a role in either the migration of Marx line/mucocutaneous junction or ARMGD. If hyperkeratinization played a role, then one might expect a posterior migration of the mucocutaneous junction, and not a movement away from the gland orifice.

### Effects of desiccating stress on meibomian gland function

Over the past decade a mouse model of desiccating stress has become widely popular for assessing the underlying mechanism of ocular surface inflammation associated with dry eye and potential therapeutic treatments [38, 39]. While studies have focused on changes in the conjunctival and corneal ocular surface, a recent report has evaluated the changes in the meibomian gland in mice exposed to the desiccating stress environment [40]. In this study, immunostaining for Ki67, a marker for cell cycle entry and cell division, was used to assess meibomian gland acinar and ductal proliferation. To assess changes in lipid quality, stimulated Raman scattering (SRS) microscopy was used to image vibrational signals from specific chemical bonds associated with protein (amide I) and lipid (CH_2_). SRS is a nonlinear optical technique that is non-destructive and non-invasive and can be used to identify specific chemical vibrational signals in cells, tissues or tissue sections that can later be stained to more precisely localize chemical patterns, particularly lipids (for details of SRS imaging please references [40, 41]).

**Fig. 4:**
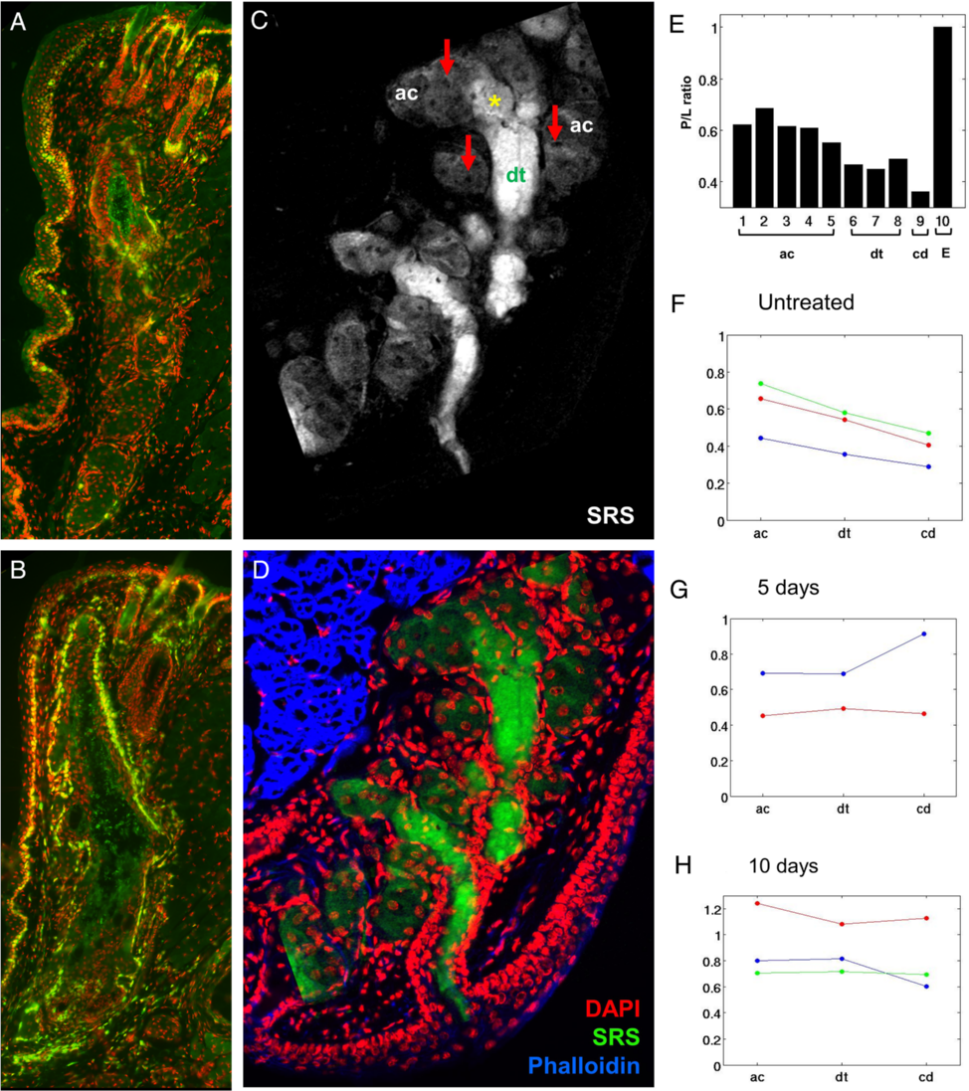
Effects of desiccating stress on meibomian gland cell proliferation (**a** and **b**) and lipid quality (**c**-**h**). Cryosections stained for the cell cycling marker, Ki67 (*green*) of eyelids from normal mice (**a**) and mice exposed to 10 days of desiccating stress (**b**). SRS microscopy of lipid within cryosections from eyelids of normal mice (**c**) and the overlay (**d**) with immunostaining for nuclei (DAPI, red), and actin (Phalloidin, blue) to identify acini (ac, arrows) and ductules (dt). **e**, Graph of protein to lipid ratio from different regions of the gland (numbered 1–10), including acini (ac), ductule (dt) central duct (cd), and extracellular matrix (**e**). **f**-**h**, graph of protein to lipid ratio for control mice (**f**) and mice exposed to 5 days (**g**) and 10 days (**h**) desiccating stress (different colors represent different mice. Note that in the normal gland there is low glandular proliferation (**a**) and a gradual decreasing protein to lipid ratio moving from the acini to the central duct (**e** and **f**). By comparison meibomian glands from mice exposed to desiccating stress show up-regulation of cell cycling (**b**) and no change in the protein to lipid ratio (**g** and **h**)

As shown in Fig. 4, meibomian glands from normal mice show a low rate of proliferation with less than 20 % of the cells appearing to be cycling as detected by immunostaining for Ki67 (a, green). By comparison, meibomian glands from mice exposed to desiccating stress show a significant increase in the number of cells that are cycling, averaging 64.4 and 66,6 % after 5 and 10 days exposure (*p* < 0.005). This finding indicates that meibomian glands are affected in the desiccating stress model, and show a marked up-regulation of cell proliferation, suggesting that there is a compensatory increase in lipid production in response to the environmental stress. Probing of tissue sections from normal eyelids using SRS microscopy (c) followed by staining for actin (d, Phalloidin) and nuclei (d, DAPI) showed lipid signals within the gland that appeared weaker in regions identified as acini (ac, arrows) and much stronger in regions identified as ducts (dt). Interestingly, acinar regions all had a high protein to lipid ratio that gradually decreased moving from the ductule to the central duct to the orifice of the gland (e). This finding suggests that lipid synthesized by the meibomian gland undergoes a maturation process where protein is gradually and continually removed from the lipid prior to exiting the orifice of the gland.

While the protein to lipid ratio in meibomian glands from control mice showed a decreasing ratio from the acini to the central duct (f), meibomian glands from mice exposed to desiccating stress for 5 days (g) and 10 days (h) showed no decrease from acini to central duct, and in some cases showed increased protein (g, blue line). This finding suggests that increased cell proliferation, acinar differentiation, and cell turnover induced by the desiccating stress model may overwhelm the ability of the gland to remove protein from the lipid prior to expression from the gland. Retention of protein most likely will have a profound effect on the lipid quality and fluidity. Recent studies assessing the surface pressure of meibomian gland lipids using a Lagmuir trough indicate that incorporation of purified keratin proteins into the lipid increases the surface pressure above that of normal meibomian gland lipid [42]. The authors postulate that a 10 % increase in keratin proteins as reported by Ong et al. [31] would make the lipid more rigid and subject to fracture if incorporated into the lipid layer of the tear film.

Importantly, these new findings suggest alternative mechanisms for the development of MGD other than ductal hyperkeratinization and obstruction. First, environmental stress or other disease mechanisms leading to increased meibomian gland duct and acinar turnover may lead to increased protein and desquamated cells and cellular debris in the central duct altering the quality and fluidity of the lipid, and potentially leading to mechanical obstruction. Such a hypothesis was originally proposed by Korb and Henriquez based on the presence of squamous cells and debris within MGD excreta [25]. This is a more likely explanation than the putative hyperkeratinization and ductal plugging, particularly since contact lens wearing alters the aqueous evaporative dynamics of the ocular surface and may lead to a similar stress response observed in the EDED mouse model. Although, secondary, inflammation-induced hyperkeratinization of the duct caused by the development of dry eye cannot be ruled out as a consequence of these environmentally induced changes in gland function and lipid quality.

Secondly, repeated stress-induced hyperproliferation of the meibomian gland may lead to early exhaustion of the progenitor or stem cell population within the gland that is necessary for maintenance of acinar function. Little is known about meibomian gland progenitor cells; however, repeated exposure to environmental stress, such as contact lens wear or ocular surface drying, may lead to a similar phenomenon to limbal stem cell deficiency where the gland can not replace acinar basal cells resulting in loss of acini and meibomian gland dropout as is seen in ARMGD in the mouse. With loss of acinar tissue, there also maybe an imbalance between lipid and ductal cells contributing to the excreta. This may result in an increase in the protein to lipid ratio, leading to altered lipid fluidity and a more rigid lipid on the tear film. Additionally, potential mechanical blockage of the gland may occur as seen in ARMGD with the epithelial plugging from ductal epithelial cells.

Finally, nothing is known about how protein is removed from the lipid once the acinar cells break down and release lipid. It is possible that this degradation pathway specifically involves the acinar cell, and thus as acinar tissue is lost, the ability to degrade protein in the duct would be compromised. Additionally, regulatory mechanisms controlling protein degradation in the duct may also be an alternative pathologic target for increasing the protein to lipid ratio and altering lipid fluidity and stiffness. Together these alternative mechanisms for the development of MGD seem more possible than that of hyperkeratinization of the orifice of the gland leading to obstruction, dilation and disuse atrophy.

## Summary

In this review we have critically evaluated the proposed pathogenetic mechanism for MGD involving hyperkeratinization of the meibomian gland duct leading to obstruction, cystic dilation and "disuse" atrophy of the meibomian gland. In considering this model we have noted several, relevant clinical and experimental findings that are not entirely consistent, particularly regarding the differences in the meibographic and biochemical findings in hyperkeratinization models compared to clinical MGD. New information on age-related changes in the meibomian gland also suggest alternative mechanism for the development of MGD and include the lack of evidence that hyperkeratinization plays a role in ARMGD, the finding that in both mouse and humans there is an anterior displacement of the mucocutaneous junction and loss of fully keratinized epithelium around the meibomian gland orifice, and the finding the desiccating stress induces proliferative changes in the gland leading to retention of protein the meibomian gland lipid that could alter lipid fluidity and stiffness. While these new findings need to be confirmed and extended, they strongly suggest alternative mechanisms to hyperkeratinization that may play a major role in the development of blepharitis-associated MGD.
